# Baptisia in Hysteria

**Published:** 1887-04

**Authors:** G. W. Sherbino

**Affiliations:** Abilene, Texas


### BAPTISIA IN HYSTERIA.

G. W. SHERBINO, ABILENE, TEXAS.

On the day of the election I was called to make a physical examination of chest and lungs, but I soon found her unfit for anything of the kind that day. Her doctor was leaving just as I was hitching my horse ; he could do nothing to relieve her unless she took a thorough course of treatment. I was kindly asked if I could do anything to relieve her.

She gave the following symptoms: She was weeping and sobbing, said her heart had quit beating, and she had difficulty in breathing; her hands and feet were cold, and she said they were dead; there was no feeling in them; her arms were numb, and it extended down into her hands; she felt as though she would be paralyzed. She kept rubbing her hands all the time; her tongue felt swollen too large, also her hands had the same feeling. (*Aethus.*) The pillow felt hard to her head; pain from the vertex down the back of the head, this pain extending down the spine, sometimes the whole length of it. She could not think; ideas confused; kept rubbing her forehead with her hands. She was very restless, was in constant motion all the time, rolling her head from side to side. She wanted to die, she did not want to live, she had nothing to live for.

℞ Baptisia$^{45m}$, Fincke, one dose. S. L. in water every ten minutes. In one hour I took my hat and left for home. From this I commenced giving her treatment; I thought I had the remedy. I spent a week looking up mind symptoms on Nux mos., and thought I had the *red string* this time. She got a dose in the morning about seven P. M.; was sent for; got there in a hurry. Husband told me he had given a dose of Bromedia; *he* did not know what else to do. "If you did not know what to do, why do anything but send for me?" "Well, Doctor, my wife must have relief, she is unconscious, does not know a thing." I asked how she was taken, and he said with the same old train of symptoms as before, pain on top of the head. And I found the symptoms were the same as before at the commencement of the attack on the previous occasion, only she was unconscious. She would catch her breast and act as though she wanted to tear it open, then she would act like one dying. "I am choking, I will die if I do not get more air!" I would raise the window; that seemed to relieve her. Pulse this time was one hundred and twenty, very full and strong; before it was fifty-six, and the temperature ninety-seven degrees; face looked swollen, and she looked as though she had been on a spree, face red. I gave a dose of Baptisia$^{45m}$ and S. L. every ten minutes. The spells came on in paroxysms. This time she seemed to be dying, but I had her pulse, and knew she was not. They did not ask me any questions, and I did not answer any, but at this moment all in the house were wild with excitement. The husband said, "Telephone Dr. G." I thought that it was about time to find my hat. I told them I would withdraw from the case, but she came to by this time and said, "Doctor, please don't go." So I sat down. As the other doctor came in I was giving a teaspoonful of S. L. He looked the case over, and she told him if he knew me as well as she did he would like me; he straightened himself up, said he had nothing in particular for me or against me. He was called into the other room, and I kept giving the S. L.

She would not take his medicine, so I kept giving the S. L. That was a very profitable council. I shall never forget it, for he did not even ask me what I was giving. He called it congestion, and if she did not get relief she might die in one of them. She has not had any more of them, although there has been premonitory symptoms several times, but a dose or two put them to flight.

I report this case for the benefit of the younger members, as such cases are sometimes troublesome. She says she can tell by her own feelings when she is going to have one of these nervous spells; circulation stops, sensation as though the heart would stop, or an imperceptible motion; features drawn, "purple linea-nasalis;" aching numbness, not a tingling, like Acon.; a dumbness, as they call it, want of sensibility; can think of nothing; by the time she tries to tell what she wants the idea is gone. First case I have ever seen cured by this remedy. Always had Morphine before; this time she did not need it.

